# Mitochondrial PCGs Provide Novel Insights into Subspecies Classification, Codon Usage and Selection of *Cervus canadensis* Distributed in Qinghai and Gansu, China

**DOI:** 10.3390/ani15101486

**Published:** 2025-05-20

**Authors:** Shiwu Dong, Lixin Tang, Sukun Yang, Xu Chen, Yang Feng, Xinhao Wang, Weilin Su, Xiumei Xing

**Affiliations:** 1 State Key Laboratory for Molecular Biology of Special Economic Animals, Institute of Special Animal and Plant Sciences, Chinese Academy of Agricultural Sciences, Changchun 130112, China; dongshiwu2017@126.com (S.D.); tanglixin1217@163.com (L.T.);; 2 College of Animal Science and Technology, Gansu Agricultural University, Lanzhou 730070, China; 3 Guangdong Chimelong Group, Guangzhou 511400, China

**Keywords:** *Cervus elaphus*, *Cervus canadensis kansuensis*, phylogeny, mitochondrial characteristics, codon usage, natural selection

## Abstract

Although *Cervus elaphus* (Linnaeus, 1758) has been well studied, the subspecific taxonomy of *Cervus canadensis* populations in Qinghai and Gansu, China, is still controversial, and the mitochondrial characteristics of *Cervus elaphus* (Linnaeus, 1758) remain incompletely understood. We assembled 89 mitogenomes of *C. canadensis* from five geographical populations across Qinghai and Gansu. Phylogenetic analysis confirmed that the 89 individuals are taxonomically classified as *C. c. kansuensis*. Nucleotide compositions showed a higher abundance of adenine and cytosine compared to guanine and thymine in both complete mitogenomes and mitochondrial PCGs. Codon usage analysis revealed a strong preference towards A-ending codons (68.04% of over-represented codons, RSCU > 1.6) in mitochondrial PCGs, with systemic avoidance of G-ending codons (53.30% of unused codons, RSCU = 0). The CAMs of 13 PCGs are reported for the first time. Furthermore, the ENC plot showed that the codon usage of all PCGs was biased except for gene *ATP8*. The PR2 bias plot showed that gene *ND6* exhibited bias towards T3 and G3, whereas the other genes preferred A3 and C3. Both the ENC-plot and PR2 bias plot suggested that natural selection played an important role in the forces driving codon usage bias in mitochondrial PCGs. Our results demonstrate the subspecific status of *C. canadensis* distributed in Qinghai and Gansu as *C. c. kansuensis*, and provide insights into the mitochondrial characteristics of *C. c. kansuensis*. The mitogenome sequences assembled in this study provide valuable data for further understanding of the *Cervus elaphus* (Linnaeus, 1758) mitogenome.

## Simple Summary

This study aimed to clarify the taxonomic status of *Cervus canadensis* distributed in Qinghai and Gansu, China, and to characterize the mitogenome of the populations. We analyzed 89 individuals of *Cervus canadensis* from five geographic populations in Qinghai and Gansu. Phylogenetic trees grouped the 89 individuals into the subspecies *C. c. kansuensis*. Analyses of mitochondrial PCGs revealed the biases of nucleotide composition and codon usage in mitogenomes of these populations. Furthermore, our findings confirmed that natural selection plays a critical role in shaping these biases. This work provides the first comprehensive analysis of mitochondrial characteristics in *C. c. kansuensis*, establishing basic data for further studies on mitogenomes of *Cervus elaphus* (Linnaeus, 1758).

## 1. Introduction

In past decades, *Cervus elaphus* (Linnaeus, 1758) has been a subject of prolonged taxonomic debate, attracting widespread attention from researchers [1,2,3,4,5,6]. The currently acknowledged taxonomic consensus is that the genus *Cervus* comprises three red deer species: *Cervus elaphus*, *Cervus canadensis* and *Cervus hanglu* [6,7,8,9]. Following the consensus, there are seven subspecies of *C. canadensis* distributed in China (*C. c. wallichi*, *C. c. macneilli*, *C. c. kansuensis*, *C. c. songaricus*, *C. c. sibiricus*, *C. c. xanthopygus* and *C. c. alashanicus* (also named as *C. c. alxaicus*)) [7,10,11,12,13], of which, *C. c. kansuensis* is distributed across Qinghai, Gansu, and northern Sichuan, and *C. c. macneilli* is endemic to western Sichuan and eastern Xizang. However, the IUCN Red List of Threatened Species (https://www.iucnredlist.org) recognizes only five subspecies of *C. canadensis* distributed in China (*C. c. wallichi*, *C. c. macneilli*, *C. c. sibiricus*, *C. c. xanthopygus* and *C. c. alashanicus*), wherein populations from Qinghai and Gansu are taxonomically grouped under *C. c. macneilli*. Therefore, further research is needed to clarify the subspecies status of *C. canadensis* distributed in Qinghai and Gansu.

A mitogenome is characterized by its small size, conserved structure, rapid evolutionary rate, maternal inheritance and exceptionally limited recombination [14,15,16,17,18], and they have been widely used in studies of origin and evolution, genetic diversity and phylogeny [3,6,7,9,19]. Generally, the complete mitogenome of vertebrates is circular and contains 22 tRNA genes, 13 protein-coding genes (PCGs), 2 rRNA genes and 1 control region [20]. Mitochondrial protein-coding sequences have been identified not only as essential tools for studying mitochondrial gene expression and functional regulation but also as valuable resources in characterizing mitochondrial features [21,22]. Previous studies have shown that the mitogenome of *Cervus elaphus* (Linnaeus, 1758) exhibits typical genome organization, comprising 37 genes and one control region [12,13,23,24,25,26,27,28,29]. However, few studies on the mitogenome of *Cervus elaphus* (Linnaeus, 1758) have been carried out, and its characteristics remain incompletely elucidated.

In this study, we collected 89 *C. canadensis* samples from five geographic populations across Qinghai and Gansu, China, and successfully assembled their complete mitogenomes. To clarify the subspecific status of these populations, phylogenetic trees were constructed using mitochondrial sequences under multiple schemes. Then, a series of analyses based on mitochondrial PCGs were performed to investigate the mitochondrial characteristics. Our results not only illustrated the subspecific status of *C. canadensis* distributed in Qinghai and Gansu, but also provided novel insights into the characteristics of mitochondrial genes in *C. c. kansuensis*. Furthermore, the 89 complete mitogenomes generated in this study significantly expand the mitochondrial genomic database for *Cervus elaphus* (Linnaeus, 1758), providing an essential data resource for future phylogenetic analysis and the mitochondrial characterization of *Cervus elaphus* (Linnaeus, 1758).

## 2. Materials and Methods

### 2.1. Sample Collection, DNA Extraction and Sequencing

**Figure 1:**
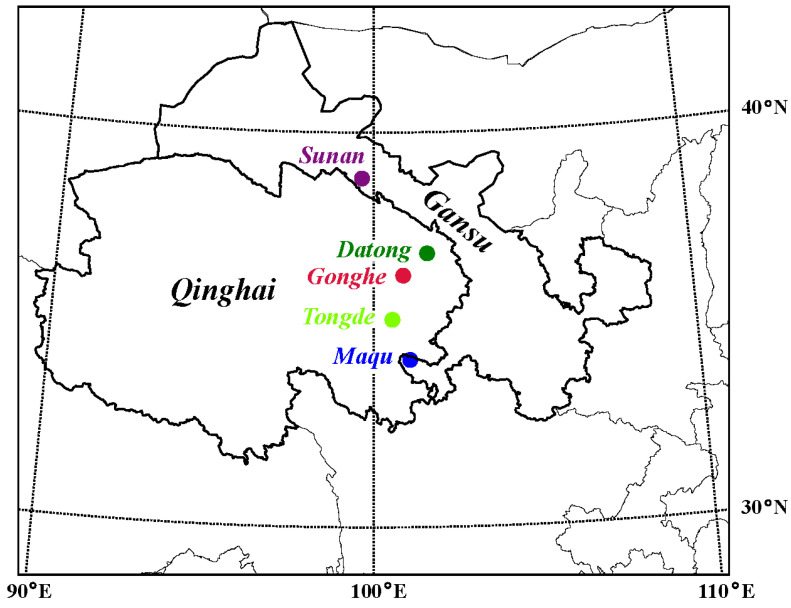
Locations of the sampled *Cervus canadensis* populations.

Overall, 89 samples of *C. canadensis* from five geographic populations in Qinghai and Gansu, China, were collected (Figure 1): 23 from Gonghe county (Gonghe), 20 from Sunan Yugur Autonomous County (Sunan), 30 from Maqu county (Maqu), 10 from Tongde county (Tongde) and 6 from Datong Hui and Tu Autonomous County (Datong). Among the 89 samples, 23 frozen velvet antlers (from Gonghe) and 26 ossified antlers (20 from Sunan, 6 from Tongde) were obtained from local farmers; the remaining 40 intravenous blood samples (10 from Datong, 30 from Maqu) were collected from anesthetized deer (Table 1). All animal study protocols were approved by the Animal Care and Use Committee of the Chinese Academy of Agricultural Sciences (approval number: ISAPSAEC-2024-034). Genomic DNA was extracted for each sample with FastPure^®^ Cell/Tissue DNA Isolation Mini Kit (Nanjing Vazyme Biotech Co., Ltd., Nanjing, China) following the standard protocol. DNA was sequenced on the Novaseq 6000 Platform of Illumina (Illumina, San Diego, CA, USA) with a PE150 sequencing method. The process of DNA sequencing took place at the Tianjin Sequencing Center, Novogene Biotech Co., Ltd., Beijing, China.

### 2.2. Mitochondrial Genome Assembly and Annotation

Raw reads sequenced were filtered using fastp v0.23.2 [30] to remove unqualified reads. Then, the filtered reads were mapped to the reference sequence downloaded from NCBI (Accession Number: NC_039923; Subspecies: *C. c. kansuensis*). Read mappings were conducted by BWA v0.7.15 [31]. SAM and BAM files were processed using samtools v1.15.1 [32], including the extraction of mapped reads and the transition of BAM to Fastq. Mitogenomes were assembled using MITObim v1.9.1. MITOS v2.1.5 [33] and MitoZ v3.5 [34] was used to identify gene features. Reference data refseq89m (https://zenodo.org/records/4284483, accessed on 29 December 2023) was used for the MITOS process and the “Chordata” clade was specified for the MitoZ process.

### 2.3. Phylogenetic Analysis

In total, thirty-nine complete mitogenomes covering seven subspecies of *C. canadensis*, four subspecies of *C. elaphus*, two subspecies of *C. hanglu*, and four individuals of *C. albirostris* downloaded from NCBI were used to investigate the phylogeny of the 89 *C. canadensis* individuals. Two sequences of *Axis axis* (JN632599 and OR143296) were used as an outgroup (Table 2). Phylogenetic analyses were performed using a maximum-likelihood method under three analytical schemes [35,36]: (1) CYTB, nucleotide alignment of gene *CYTB*; (2) PCGs_nt12, nucleotide alignment of concatenated 13 PCGs with the first two codon positions; (3) PCGs, nucleotide alignment of concatenated 13 PCGs. Phylogenetic trees were calculated using IQ-TREE v2.2.2.6 [37]. The best-fit model for each alignment was estimated using the ModelFinder algorithm [38] as implemented in IQ-TREE v2.2.2.6. Branch support was evaluated with ultrafast bootstrap approximation [39] for 1000 replicates. Resultant trees were visualized using Interactive Tree of Life (iTOL, https://itol.embl.de, accessed on 12 November 2024).

### 2.4. Analysis of Mitochondrial Characteristics

Sequence alignments were performed using megacc v10.2.6 [41] with the MUSCLE Codon Alignment pattern. Nucleotide composition and the relative synonymous codon usage (RSCU) were calculated using MEGA v10.2.6 [41]. Skew values of PCGs were calculated using Formula (1) [42]:

AT-skew = (*A* − *T*)/(*A* + *T*); GC-skew = (*G* − *C*)/(*G* + *C*), (1)

where *A*, *T*, *G*, and *C* represent the nucleotide frequencies of A, T, G, and C, respectively. The values for the effective number of codons (ENCs) were calculated using CodonW v1.4.4 (https://sourceforge.net/projects/codonw, accessed on 21 January 2024). To generate the standard curve in ENC-plot, *GC*3 values from 0 to 1 with an interval of 0.001 were used to compute the corresponding expected ENC values using Formula (2) [22]:

ENC = 2 + *GC3* + 29/[*GC3*^2^ + (1 − *GC3*)^2^]. (2)

*GC3* represents the GC content of genes in its third codon positions. Parity Rule 2 (PR2) biases were analyzed using four-fold degenerate codons of PCGs. The extraction of four-fold degenerate codons and the statistics for the nucleotide composition of four-codon sequences were performed using Python (v3.8.6) scripts. A vertebrate mitochondrial genetic codon table was specified in all the related processes above.

## 3. Results and Discussion

### 3.1. Genome Composition and Organization

A total of 89 mitogenomes were assembled in this study. The length of these genomes varies from 16,428 bp to 16,450 bp, with GC content from 37.75 to 37.91%, showing a common feature of AT bias in mammalian mitogenomes [43]. The positive AT-skew values and negative GC-skew values indicated that adenine was more abundant than thymine, and cytosine was more abundant than guanine in the mitogenomes of the 89 individuals [44] (Table S1).

As previously described in several studies [12,23,25,26,27], the typical 37 genes and one control region were identified in each of the mitogenomes analyzed. The gene features were highly conserved across the 89 individuals: only 1 rRNA (*rrnL*), 2 tRNA (*trnI* and *trnH*), 1 PCG (*ND5*) and the control region exhibited a variation in length. Among the 13 PCGs, incomplete stop codons are commonly reported in gene *COX3* (TA-), *ND3* (T--/TA-) and *ND4* (T--) [12,23,25,26,27]. In this study, while the typical incomplete stop codon (T--) in gene *ND4* was observed, all 89 mitogenomes exhibited complete stop codons in both gene *COX3* (TAG) and *ND3* (TAG) (Table 3).

### 3.2. Phylogeny of Cervus canadensis

**Figure 2:**
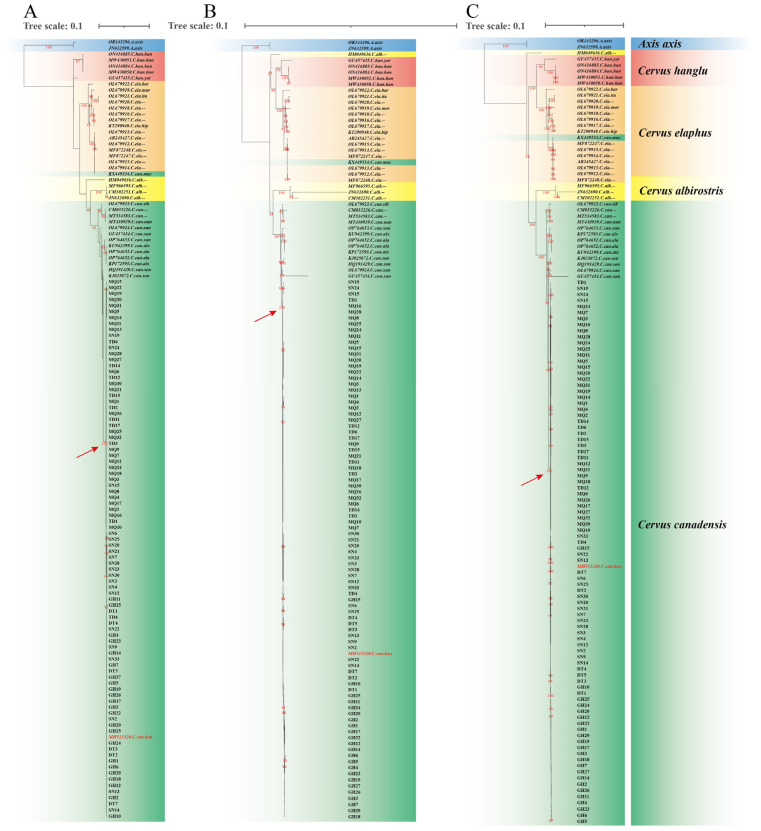
Phylogenetic trees constructed using three schemes: (**A**) CYTB; (**B**) PCGs_nt12; (**C**) PGCs. Different species are colored by different background colors. Sequences obtained from NCBI are indicated in italics. Red arrows point to 100% bootstrap supported clades formed by 89 mitogenomes and *C. c. kansuensis*. “--” denotes the subspecies information is not applicable. The sequence MH513320 is highlighted in red. Bootstrap values ≥ 80 are shown in red along the branches. Red arrows point to clades containing both the 89 sampled individuals and sequence MH513320. Sample prefixes denote collection locations: SN (Sunan), DT (Datong), GH (Gonghe), TD (Tongde), and MQ (Maqu).

Mitochondrial sequences have been extensively used for phylogenetic studies of Cervinae [2,4,6,8,45], with the currently acknowledged taxonomy of *Cervus elaphus* (Linnaeus, 1758) being proposed on the basis of mitochondrial phylogenetic analyses [6]. In this study, we performed phylogenetic analysis using 41 complete mitogenomes downloaded from the NCBI and 89 newly assembled *C. canadensis* mitogenomes. According to the Bayesian information criterion, the best-fit models for CYTB, PCGs_nt12 and PCGs were HKY + F + G4, TPM2u + F + R2 and TPM2u + F + R2, respectively. The phylogenetic trees constructed using three schemes produced basically the same topologies (Figure 2). The 89 mitogenomes formed a well-supported clade (100% bootstrap) with *C. c. kansuensis* (MH513320), as indicated by red arrows, while being distinct from *C. c. macneilli* (KX449334). This result suggests the classification of 89 sampled individuals from five geographic populations across Qinghai and Gansu as *C. c. kansuensis*. The sequence KX449334 was derived from western Sichuan, the typical distribution area of *C. c. macneilli* [10,11]. Both our results and prior research [7] demonstrate clear different phylogeny between KX449334 and MH513320, supporting *C. c. kansuensis* and *C. c. macneilli* as different subspecies. However, future studies incorporating more comprehensive sampling across their distribution regions and additional molecular markers would further validate this taxonomic conclusion.

### 3.3. Nucleotide Bias of Mitochondrial Protein-Coding Genes

**Figure 3:**
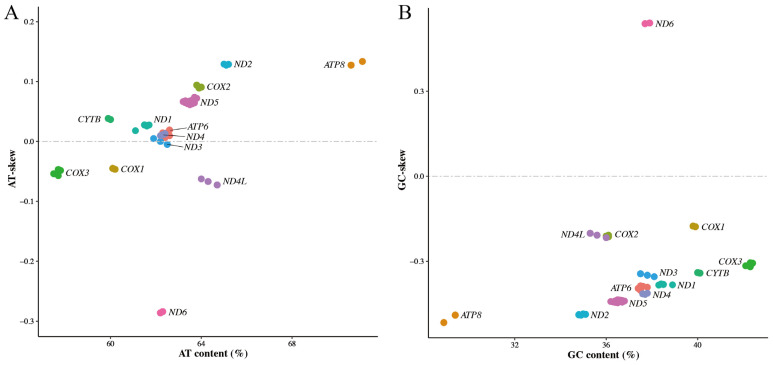
Skewness plots of 13 mitochondrial protein-coding genes of *Cervus canadensis*. (**A**) AT-skew plot. (**B**) GC-skew plot. Different colors represent different genes.

The skews of complete mitogenomes are useful to display the discrepancy of nucleotide composition among different species [44,46], while the skews of mitochondrial genes can reflect the bias in nucleotide composition of different genes in a species [43,47,48]. Here, the nucleotide composition and skews of mitochondrial PCGs were calculated. The skewness plots (Figure 3) were plotted using AT-skew (GC-skew) against the AT (GC) content [44]. The nucleotide composition results showed the GC contents were from 28.9% to 42.4% in 13 PCGs, and the AT contents were from 57.5% to 71.1%. The biases towards AT content occurred in each of the PCGs, similarly to what was previously reported for other species [43]. Moreover, our AT-skew plot (Figure 3A) highlighted eight PCGs (*ATP8*, *ND2*, *COX2*, *ND5*, *CYTB*, *ND1*, *ATP6* and *ND4*) having positive skew values, and four PCGs (*ND6*, *ND4L*, *COX3* and *COX1*) with negative skew values. The GC-skew plot (Figure 3B) showed that all PCGs had negative skew values except for gene *ND6*. These results are similar to those of the skewness of mitochondrial PCGs in Teressa goat [43] and India wild pig [47], where most of the mitochondrial PCGs had positive AT-skew values and negative GC-skew values. The positive AT-skew values and negative GC-skew values in most PCGs demonstrated the higher abundance of adenine and cytosine than guanine and thymine in mitochondrial PCGs of *C. c. kansuensis*.

### 3.4. Codon Usage of Mitochondrial Protein-Coding Genes

Codon usage patterns (CUPs) refer to the non-random selection preferences exhibited by organisms for synonymous codons during the translation process [49,50]. The RSCU is helpful for detecting the CUPs for all synonymous codons in individual genes [51]. RSCU value is defined as the ratio of the observed frequency of a codon to its expected frequency, and is independent of the sequence length or amino acid composition [52]. Codons with an RSCU value greater than 1.6 or less than 0.6 are, respectively, regarded as over-represented or under-represented, while the others are considered unbiased or used randomly [53]. Notably, an RSCU value of 0 indicates complete absence of codon usage within individual genes, a phenomenon defined as codon aversion [54,55]. A set of codons unused by an individual gene is defined as codon aversion motif (CAM) [56]. Both the CUPs and CAMs are phylogenetically conserved, and so can be used in phylogenomic studies in the future [54,56]. Here, the mean RSCU values of each mitochondrial PCG in 89 individuals were calculated. The results revealed substantial codon usage biases among the 13 PCGs, with 63.82% of codons showing non-random CUPs: 25.48% unused codons (RSCU = 0), 15.02% under-represented codons (0 < RSCU < 0.6) and 23.32% over-represented codons (RSCU > 1.6). Strikingly, G-ending codons dominated the unused category, with a percentage of 53.30%, while A-ending codons were preferred among over-represented codons (68.04%). In contrast, U-ending and C-ending codons exhibited minor bias, accounting for 76.41% of unbiased codons (Table 4).

**Figure 4:**
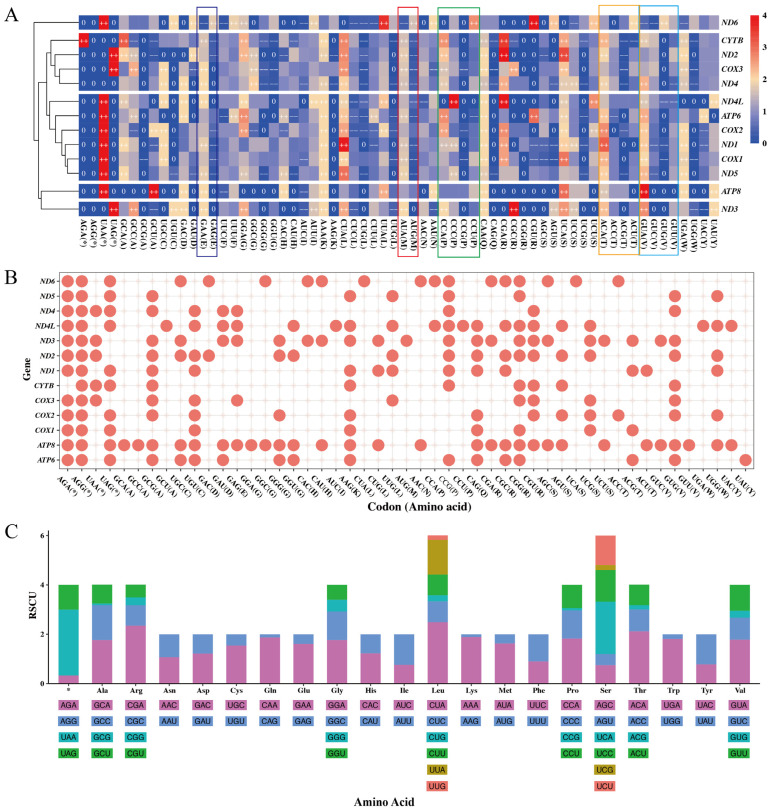
Codon usage statistics of the *Cervus canadensis* mitochondrial genome. (**A**) CUPs of 13 mitochondrial PCGs. Colors from blue to red represent RSCU values from small to large. “++” denotes RSCU value > 1.6, “−−” denotes RSCU value < 0.6 and “0” denotes RSCU value = 0. Colored rectangles highlight codon usage bias specific to gene *ND6*. (**B**) CAMs of 13 mitochondrial PCGs. Codons unused in each PCG are marked as red points. (**C**) Statistics of codon usage for synonymous codons of each amino acid and stop codon (*) in combination of 13 mitochondrial PCGs. Different colors denote different codons.

To better understand the CUPs of 13 mitochondrial PCGs in *C. c. kansuensis*, a hierarchical clustering heatmap was drawn using the mean RSCU value of each codon in each PCG (Figure 4A). The hierarchical clustering heatmap revealed different codon usage bias among the 13 PCGs. Notably, the gene ND6 preferentially used AUG as its start codon, whereas AUA was preferred in most other genes (highlighted by red rectangle). Similar codon preference patterns were observed for amino acid E (Glu), P (Pro), T (Thr), and V (Val), as indicated by colored rectangles in Figure 4A. According to the hierarchical clustering result, the CUPs of 13 PCGs can roughly be divided into five groups: (1) *ND6*; (2) *ATP8*; (3) *ND3*; (4) *CYTB*, *ND2*, *COX3* and *ND4*; and (5) *ND4L*, *ATP6*, *COX2*, *ND1*, *COX1* and *ND5*. Furthermore, following the definition of CAM [56], the CAM of a PCG comprises all its codons with an RSCU equal to 0. We visualized the CAMs of the 13 PCGs based on mean RSCU values (Figure 4B). The CAMs of 13 PCGs in *C. c. kansuensis* consisted of 49 codons encoding 19 amino acids and 4 termination codons. The number of unused codons across the 13 CAMs varied from 9 to 32.

To investigate CUPs at the amino acid level, we calculated the mean RSCU values for the concatenated 13 PCGs of 89 individuals. As shown in Figure 4C, the CUPs of amino acids in *C. c. kansuensis* mitochondrial PCGs revealed that the most preferred stop codon was UAA, and the most preferred start codon was AUA. For amino acids with synonymous codons ending in A or G (Glu, Lys, Met, Gln, and Trp), there was a strong preference for A-ending codons. In contrast, amino acids with synonymous codons ending in C or U (Cys, Asp, Phe, His, Ile, Asn and Tyr) exhibited random CUPs with no clear preference (0.6 < RSCU < 1.6). However, Cys was an exception, where the codon UGU was under-represented (RSCU = 0.46).

### 3.5. Effective Number of Codons

The ENC, with values ranging from 20 to 61, exhibits a negative correlation with codon usage bias [35,57]. The lower the ENC value, the stronger the codon usage bias of the gene. A gene with an ENC value less than 35 is considered to have a strong codon usage bias [20,35]. Here, the ENC values of 13 mitochondrial PCGs in 89 individuals were calculated. Among the 13 PCGs, the gene ATP8 showed the highest ENC values from 60.95 to 61, indicating negligible codon usage bias. With the exception of the gene *ATP8* and *ND4L*, the ENC values of remaining genes were distributed from 36.34 to 45.32, signifying weak codon usage bias in mitochondrial PCGs across *C. c. kansuensis* populations.

**Figure 5:**
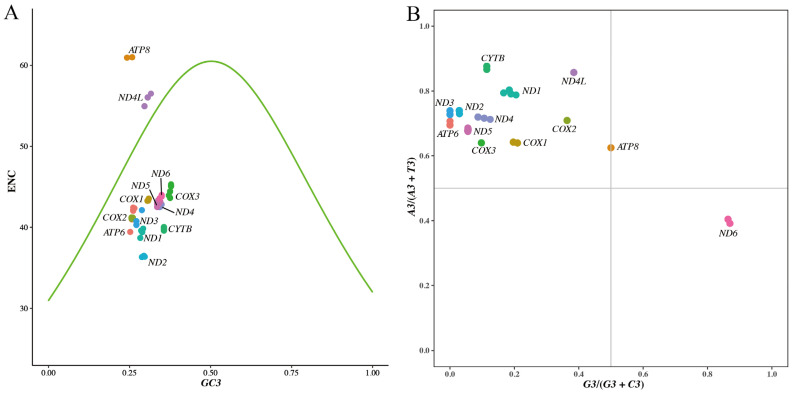
Codon bias distribution of *Cervus canadensis* mitochondrial genome. (**A**) ENC-plot of 13 mitochondrial PCGs. ENC denotes value of effective number of codons. *GC3* denotes GC content at the third codon position. (**B**) PR2 biases at third codon position of four-codon sequences in 13 mitochondrial PCGs. *A3*, *T3*, *G3* and *C3* represent nucleotide (A, T, G and C) contents at the third codon position.

In order to further evaluate whether the mutation pressure and natural selection have influenced the codon usage bias, an ENC plot was drawn using ENC values against *GC3*s [21,22,35,57,58,59]. When the ENC value of a gene falls near the standard curve in the ENC plot, mutation pressure was thought to be the only factor affecting its third-position bases of codons. If the ENC value falls far below the standard curve, the codon bias of that gene was affected by natural selection [22,51,53,57]. In our ENC plot (Figure 5A), most genes clustered below the standard curve, suggesting that natural selection was the primary factor affecting their codon usage bias. The points of the gene *ND4L* were positioned near the standard curve, suggesting its codon usage bias was mainly influenced by mutation pressure. These results collectively demonstrate that the codon usage bias of mitochondrial PCGs in *C. c. kansuensis* populations is affected more by natural selection than mutation.

### 3.6. PR2 Bias Analysis

PR2 describes the base composition rule in double-stranded DNA; the frequencies of A and T are approximately equal within a single strand, as are those of G and C, when mutation and selection are equally effective on both strands [60]. PR2 biases at the third codon position in four-codon sequences are considered to be effective indices to estimate the forces that drive deviation from neutral mutations in individual genes [53,58]. Points of genes should fall in the center of the PR2 bias plot, where both coordinates are 0.5, if codon usage bias is caused only by mutation pressure [58,61]. According to the vertebrate mitochondrial genetic codon table, there are 24 four-fold degenerate codons encoding 6 amino acids (Ala, Arg, Gly, Pro, Thr and Val). We extracted the four-codon sequences for each mitochondrial PCG from the 89 mitogenomes and analyzed PR2 biases at their third codon positions. The PR2 bias plot (Figure 5B) revealed that all PCG data points were distributed in the second quadrant, except for the genes *ATP8* and *ND6*, indicating that 11 of 13 mitochondrial PCGs in *C. c. kansuensis* exhibit codon usage bias preferring A and C in the third codon position. The GC-bias values (*G3*/(*G3* + *C3*)) of gene *ATP8* were 0.5, indicating no bias to G or C at its third codon position. However, the AT-bias values (*A3*/(*A3* + *T3*)) exceeded 0.5, suggesting its preference for A. The data points of the gene *ND6* fell in the fourth quadrant, relatively close to the *X*-axis but distant from the *Y*-axis, suggesting that the gene *ND6* had a strong preference for G and a weak preference for T. These observed biases in mitochondrial PCGs suggest that mutation pressure is not the only power affecting mitochondrial codon usage bias of *C. c. kansuensis*, which is consistent with the results of the ENC-plot analysis.

## 4. Conclusions

In this study, we assembled 89 complete mitogenomes of *Cervus canadensis* from five geographic populations across Qinghai and Gansu, China. Phylogenetic analysis confirmed that the five *C. canadensis* populations are taxonomically classified as *C. c. kansuensis*. Our analyses revealed that the nucleotide composition is biased towards AT in mitochondrial PCGs of *C. c. kansuensis*, and the adenine and cytosine is preferred over guanine and thymine. Moreover, the distinct codon usage patterns and codon aversion motifs observed among the 13 PCGs, along with the preferences of amino acids for synonymous codons, indicate significant codon usage bias in mitochondrial PCGs of *C. c. kansuensis*. Both the ENC plot and PR2 bias plot demonstrated that natural selection plays a critical role in shaping these biases. This study provides evidence for the subspecific classification of *C. canadensis* populations in Qinghai and Gansu, China, while offering novel insights into the characteristics of mitochondrial PCGs in *C. c. k ansuensis*. The complete mitogenomes generated in this research provide a valuable resource for further understanding the mitochondrial characteristics of *Cervus elaphus* (Linnaeus, 1758).

## Figures and Tables

**Table 1:** Number and type of samples collected from different geographic locations.

Location	Longitude	Latitude	Number	Sample Type
Sunan	99.6	38.8	20	ossified antler
Datong	101.7	36.9	10	intravenous blood
Gonghe	100.9	36.4	23	frozen velvet antler
Tongde	100.6	35.3	6	ossified antler
Maqu	101.1	34.2	30	intravenous blood

**Table 2:** Mitochondrial sequences downloaded from NCBI.

Accession Number	Length (bp)	Species	Subspecies	Location
JN632599	16,349	*Axis axis*	--	--
OR143296	16,351	*Axis axis*	--	--
CM102251	16,474	*C. albirostris*	--	--
HM049636	16,478	*C. albirostris*	--	--
JN632690	16,512	*C. albirostris*	--	HT *
MF966595	16,473	*C. albirostris*	--	China
CM033226	16,429	*C. canadensis*	--	America
GU457434	16,416	*C. canadensis*	*xanthopygus*	--
HQ191429	16,419	*C. canadensis*	*songaricus*	--
KJ025072	16,419	*C. canadensis*	*songaricus*	--
KP172593	16,428	*C. canadensis*	*alxaicus*	China
KU942399	16,503	*C. canadensis*	*alxaicus*	China
KX449334	16,350	*C. canadensis*	*macneilli*	China
MH513320	16,430	*C. canadensis*	*kansuensis*	China
MT430939	16,428	*C. canadensis*	*nannodes*	Republic of Korea
MT534583	16,428	*C. canadensis*	--	Republic of Korea
OL679923	16,428	*C. canadensis*	*sibiricus*	Russia
OL679924	16,353	*C. canadensis*	*xanthopygus*	Russia
OP764651	16,430	*C. canadensis*	*alashanicus*	China
OP764652	16,436	*C. canadensis*	*alashanicus*	China
OP764653	16,430	*C. canadensis*	*xanthopygus*	China
AB245427	16,357	*C. elaphus*	--	New Zealand
KT290948	16,354	*C. elaphus*	*hippelaphus*	Hungary
MF872247	16,357	*C. elaphus*	--	Denmark
MF872248	16,357	*C. elaphus*	--	Denmark
OL679912	16,351	*C. elaphus*	--	Poland
OL679913	16,351	*C. elaphus*	--	Poland
OL679914	16,352	*C. elaphus*	--	Poland
OL679915	16,352	*C. elaphus*	--	Poland
OL679916	16,355	*C. elaphus*	--	Poland
OL679917	16,355	*C. elaphus*	--	Poland
OL679918	16,354	*C. elaphus*	--	Poland
OL679919	16,350	*C. elaphus*	*maral*	Iran
OL679920	16,351	*C. elaphus*	--	Poland
OL679921	16,351	*C. elaphus*	*italicus*	Italy
OL679922	16,353	*C. elaphus*	*barbarus*	Tunisia
GU457435	16,351	*C. hanglu*	*yarkandensis*	--
MW430050	16,354	*C. hanglu*	*hanglu*	India
MW430051	16,354	*C. hanglu*	*hanglu*	India
ON416884	16,351	*C. hanglu*	*hanglu*	India
ON416885	16,351	*C. hanglu*	*hanglu*	India

--: The information is not applicable. *: Haute Touche Animal Park (Indre, France) [40].

**Table 3:** Gene features of 89 assembled genomes.

Gene (Anticodon)	Length (bp)	Strand	Start Codon	Stop Codon	Gene (Anticodon)	Length (bp)	Strand	Start Codon	Stop Codon
*trnF* (gaa)	69	+			*trnK* (uuu)	69	+		
*rrnS*	956	+			*ATP8*	201	+	ATG	TAA
*trnV* (uac)	67	+			*ATP6*	681	+	ATG	TAA
*rrnL*	1574 ^a^	+			*COX3*	804	+	ATG	TAG
*trnL* (uaa)	75	+			*trnG* (ucc)	69	+		
*ND1*	957	+	ATG	TAA	*ND3*	357	+	ATA	TAG
*trnI* (gau)	69 ^b^	+			*trnR* (ucg)	69	+		
*trnQ* (uug)	72	−			*ND4L*	297	+	ATG	TAA
*trnM* (cau)	69	+			*ND4*	1378	+	ATG	T--
*ND2*	1044	+	ATA	TAG	*trnH* (gug)	69 ^c^	+		
*trnW* (uca)	68	+			*trnS* (gcu)	60	+		
*trnA* (ugc)	69	−			*trnL* (uag)	70	+		
*trnN* (guu)	73	−			*ND5*	1821 ^d^	+	ATA	TAA
*trnC* (gca)	68	−			*ND6*	528	−	ATG	TAA
*trnY* (gua)	69	−			*trnE* (uuc)	69	−		
*COX1*	1546	+	ATG	TAA	*CYTB*	1140	+	ATG	AGA
*trnS* (uga)	70	−			*trnT* (ugu)	70	+		
*trnD* (guc)	68	+			*trnP* (ugg)	66	−		
*COX2*	684	+	ATG	TAA	Control Region	522 ^e^	+		

^a^ *rrnL* with length of 1575 bp was observed in 32 individuals; ^b^ 1 exception observed with *trnI* (tRNA-Ile) and length of 70 bp; ^c^ *trnH* (tRNA-His) with length of 70 bp was observed in 7 of 89 individuals; ^d^ 2 individuals have *ND5* genes with length of 1824 bp and 1 has length of 1827 bp; ^e^ length of control region varied from 512 bp to 525 bp, with highest frequency of 522 bp (44 instances), followed by 520 bp (31 instances).

**Table 4:** Frequencies of codons ending with different nucleotide in different RSCU intervals.

RSCU Value	Number of Codons					Percentage (%)
	A-Ending ^1^	G-Ending ^2^	U-Ending ^3^	C-Ending ^4^	Total	
0	27	113	42	30	212	25.48
0~0.6	4	63	27	31	125	15.02
0.6~1.6	45	26	113	117	301	36.18
>1.6	132	6	26	30	194	23.32

^1^ Codons ending with A; ^2^ codons ending with G; ^3^ codons ending with U(T); ^4^ codons ending with C.

## Data Availability

The complete mitochondrial genomes assembled in this study are available on reasonable request from the corresponding author as the research project is conducted by a national institution.

## Supplementary Materials

The following supporting information can be downloaded at https://www.mdpi.com/article/10.3390/ani15101486/s1; Table S1: Nucleotide composition of 89 mitochondrial genomes.

## Abbreviations

The following abbreviations are used in this manuscript:

NCBI	National Center for Biotechnology Information
PCG	Protein-coding gene
IUCN	International Union for Conservation of Nature
PR2	Parity Rule 2
ENC	Effective Number of Codons
RSCU	Relative Synonymous Codon Usage
CUP	Codon Usage Pattern
CAM	Codon Aversion Motif
