# Supplementary material for: Mitochondrial PCGs Provide Novel Insights into Subspecies Classification, Codon Usage and Selection of Cervus canadensis Distributed in Qinghai and Gansu, China
Source: Animals (Basel). 2025 May 20;15(10):1486. doi: 10.3390/ani15101486 (PMC12108357; doi:10.3390/ani15101486)
Supplement: Supplementary file 1 [file animals-15-01486-s001.zip › animals-3618165-supplementary.pdf]

Supplementary Materials

Table S1. Nucleotide composition of 89 mitochondrial genomes.

Sample Name	Length (bp)	A (%)	T (%)	G (%)	C (%)	AT (%)	GC (%)	AT-skew	GC−skew
MQ10	16430	33.34	28.84	13.51	24.31	62.18	37.82	0.07	−0.29
MQ11	16430	33.34	28.86	13.51	24.28	62.2	37.8	0.07	−0.29
MQ12	16431	33.33	28.87	13.52	24.28	62.2	37.8	0.07	−0.28
MQ13	16430	33.35	28.86	13.51	24.28	62.21	37.79	0.07	−0.29
MQ14	16435	33.33	28.83	13.53	24.31	62.17	37.83	0.07	−0.28
MQ15	16429	33.36	28.87	13.5	24.27	62.23	37.77	0.07	−0.29
MQ16	16430	33.34	28.86	13.51	24.28	62.2	37.8	0.07	−0.29
MQ17	16432	33.34	28.87	13.5	24.28	62.21	37.79	0.07	−0.29
MQ18	16432	33.34	28.85	13.55	24.27	62.18	37.82	0.07	−0.28
MQ19	16433	33.34	28.85	13.49	24.32	62.19	37.81	0.07	−0.29
MQ1	16432	33.35	28.87	13.49	24.29	62.22	37.78	0.07	−0.29
MQ20	16430	33.35	28.85	13.49	24.3	62.2	37.8	0.07	−0.29
MQ21	16432	33.34	28.85	13.52	24.29	62.19	37.81	0.07	−0.29
MQ22	16429	33.36	28.85	13.49	24.3	62.21	37.79	0.07	−0.29
MQ24	16430	33.34	28.86	13.51	24.28	62.2	37.8	0.07	−0.29
MQ25	16430	33.34	28.86	13.51	24.28	62.2	37.8	0.07	−0.29
MQ26	16431	33.34	28.87	13.51	24.28	62.21	37.79	0.07	−0.29
MQ27	16430	33.34	28.84	13.51	24.31	62.18	37.82	0.07	−0.29
MQ28	16430	33.34	28.86	13.51	24.28	62.2	37.8	0.07	−0.29
MQ29	16429	33.34	28.87	13.51	24.28	62.21	37.79	0.07	−0.29
MQ2	16431	33.33	28.87	13.51	24.29	62.2	37.8	0.07	−0.29
MQ30	16435	33.34	28.87	13.51	24.28	62.2	37.8	0.07	−0.28
MQ31	16431	33.35	28.83	13.54	24.28	62.18	37.82	0.07	−0.28
MQ32	16433	33.34	28.87	13.5	24.29	62.21	37.79	0.07	−0.29
MQ3	16430	33.35	28.87	13.51	24.28	62.22	37.78	0.07	−0.29
MQ4	16432	33.34	28.87	13.49	24.3	62.21	37.79	0.07	−0.29
MQ5	16430	33.36	28.86	13.49	24.3	62.22	37.78	0.07	−0.29
MQ6	16433	33.34	28.87	13.5	24.29	62.21	37.79	0.07	−0.29
MQ7	16430	33.34	28.87	13.51	24.28	62.2	37.8	0.07	−0.29
MQ8	16430	33.34	28.86	13.51	24.28	62.2	37.8	0.07	−0.29
MQ9	16433	33.34	28.86	13.5	24.3	62.19	37.81	0.07	−0.29
GH10	16433	33.37	28.84	13.49	24.3	62.2	37.8	0.07	−0.29
GH11	16439	33.41	28.83	13.48	24.28	62.24	37.76	0.07	−0.29
GH12	16433	33.37	28.85	13.49	24.29	62.22	37.78	0.07	−0.29
GH13	16432	33.36	28.85	13.47	24.31	62.21	37.79	0.07	−0.29
GH14	16430	33.37	28.83	13.49	24.31	62.2	37.8	0.07	−0.29
GH15	16431	33.32	28.85	13.54	24.3	62.16	37.84	0.07	−0.28
GH16	16431	33.38	28.84	13.49	24.3	62.22	37.78	0.07	−0.29
GH17	16432	33.37	28.85	13.49	24.29	62.22	37.78	0.07	−0.29
GH18	16430	33.37	28.84	13.49	24.3	62.21	37.79	0.07	−0.29
GH19	16430	33.37	28.85	13.49	24.3	62.22	37.78	0.07	−0.29
GH1	16430	33.37	28.84	13.51	24.29	62.2	37.8	0.07	−0.29
GH20	16430	33.37	28.84	13.49	24.3	62.21	37.79	0.07	−0.29
GH21	16431	33.37	28.84	13.49	24.3	62.21	37.79	0.07	−0.29
GH22	16433	33.37	28.86	13.49	24.29	62.23	37.77	0.07	−0.29
GH23	16430	33.37	28.84	13.48	24.31	62.21	37.79	0.07	−0.29

GH24	16430	33.37	28.85	13.49	24.3	62.22	37.78	0.07	−0.29
GH25	16433	33.37	28.85	13.49	24.29	62.22	37.78	0.07	−0.29
GH26	16430	33.37	28.84	13.49	24.3	62.21	37.79	0.07	−0.29
GH27	16430	33.36	28.84	13.49	24.31	62.2	37.8	0.07	−0.29
GH28	16429	33.36	28.84	13.49	24.31	62.2	37.8	0.07	−0.29
GH29	16430	33.37	28.85	13.49	24.3	62.22	37.78	0.07	−0.29
GH2	16433	33.38	28.84	13.49	24.3	62.22	37.78	0.07	−0.29
GH30	16434	33.37	28.85	13.49	24.29	62.22	37.78	0.07	−0.29
GH3	16430	33.37	28.84	13.49	24.3	62.21	37.79	0.07	−0.29
GH4	16430	33.38	28.84	13.48	24.3	62.22	37.78	0.07	−0.29
GH5	16436	33.4	28.84	13.48	24.28	62.24	37.76	0.07	−0.29
GH6	16433	33.41	28.83	13.49	24.27	62.24	37.76	0.07	−0.29
GH7	16430	33.36	28.84	13.49	24.32	62.2	37.8	0.07	−0.29
GH8	16438	33.35	28.81	13.51	24.33	62.16	37.84	0.07	−0.29
GH9	16432	33.37	28.85	13.49	24.3	62.21	37.79	0.07	−0.29
SN12	16429	33.34	28.86	13.51	24.29	62.19	37.81	0.07	−0.29
SN13	16429	33.34	28.85	13.51	24.3	62.19	37.81	0.07	−0.29
SN14	16431	33.35	28.85	13.49	24.31	62.2	37.8	0.07	−0.29
SN15	16429	33.37	28.88	13.48	24.27	62.25	37.75	0.07	−0.29
SN19	16429	33.37	28.88	13.48	24.27	62.25	37.75	0.07	−0.29
SN20	16429	33.34	28.86	13.51	24.29	62.19	37.81	0.07	−0.29
SN21	16429	33.34	28.86	13.51	24.29	62.2	37.8	0.07	−0.29
SN22	16429	33.34	28.86	13.51	24.29	62.2	37.8	0.07	−0.29
SN23	16428	33.34	28.85	13.51	24.29	62.19	37.81	0.07	−0.29
SN24	16429	33.37	28.88	13.48	24.27	62.25	37.75	0.07	−0.29
SN25	16429	33.34	28.85	13.51	24.3	62.19	37.81	0.07	−0.29
SN28	16429	33.34	28.86	13.51	24.29	62.19	37.81	0.07	−0.29
SN2	16428	33.33	28.85	13.51	24.3	62.19	37.81	0.07	−0.29
SN30	16430	33.29	28.9	13.52	24.29	62.19	37.81	0.07	−0.28
SN33	16450	33.27	28.82	13.55	24.36	62.09	37.91	0.07	−0.29
SN3	16440	33.34	28.86	13.5	24.3	62.2	37.8	0.07	−0.29
SN4	16429	33.34	28.86	13.51	24.29	62.19	37.81	0.07	−0.29
SN6	16429	33.34	28.85	13.51	24.3	62.19	37.81	0.07	−0.29
SN7	16429	33.34	28.86	13.51	24.29	62.19	37.81	0.07	−0.29
SN9	16435	33.37	28.82	13.48	24.33	62.2	37.8	0.07	−0.29
DT1	16430	33.36	28.84	13.49	24.3	62.2	37.8	0.07	−0.29
DT2	16429	33.34	28.87	13.51	24.28	62.21	37.79	0.07	−0.28
DT3	16432	33.34	28.87	13.5	24.29	62.21	37.79	0.07	−0.29
DT4	16436	33.32	28.83	13.51	24.34	62.16	37.84	0.07	−0.29
DT5	16437	33.33	28.84	13.51	24.32	62.17	37.83	0.07	−0.29
DT7	16439	33.33	28.85	13.52	24.3	62.18	37.82	0.07	−0.29
TD11	16429	33.35	28.87	13.5	24.28	62.22	37.78	0.07	−0.29
